# Systematic Review and Meta-analysis of Admission Inflammatory Biomarkers for Evaluating Prognosis in Acute Type A Aortic Dissection

**DOI:** 10.1055/a-2693-4070

**Published:** 2025-10-07

**Authors:** Sarah Shirley, Alana Maerivoet, Helen L. Wright, Mark Field, Jillian Madine

**Affiliations:** 1 Department of Cardiothoracic Surgery, Liverpool Heart and Chest Hospital, Liverpool, United Kingdom; 2 Institute of Systems, Molecular and Integrative Biology, Faculty of Health and Life Sciences, University of Liverpool, Liverpool, United Kingdom; 3 Institute of Life Course and Medical Sciences, Faculty of Health and Life Sciences, University of Liverpool, Liverpool, United Kingdom

**Keywords:** acute dissection, prognostic markers, inflammation

## Abstract

Acute type A aortic dissection (ATAAD) is traumatic and life-threatening involving a split of the intima media along a variable length of the aorta from aortic root to aortic bifurcation. The pathology results in a local and systemic inflammatory process with elevated inflammatory markers observed at hospital admission. This systematic literature review aimed to compare the effectiveness of admission inflammatory markers in predicting adverse outcomes in postoperative ATAAD patients. Eligibility criteria included studies reporting postoperative outcomes or receiver operating characteristic results stratified by routine admission markers of inflammation in ATAAD patients. The study protocol was registered with PROSPERO (CRD42022366509). Following abstract and full-text screening, 79 studies were included in the analysis, with 39 included in the meta-analysis. Meta-analyses using random effects models of white blood cell count, neutrophil count, and neutrophil to lymphocyte ratio stratified by survival indicated that levels were significantly lower in survivors than nonsurvivors. The mean difference for white blood cell count was 1.51 (confidence interval [CI = 1.07, 1.95]), neutrophil count 1.50 [CI = 1.05, 1.95], and neutrophil to lymphocyte ratio 3.45 [CI = 2.50, 4.41]. Similarly, survivors had lower C-reactive protein levels than nonsurvivors (standardized mean difference = 0.5227 [CI = 0.1781, 0.8672]). Conversely, lymphocyte counts were higher in survivors than nonsurvivors (mean difference = −0.12 [CI = −0.18, −0.06]). All models had significant heterogeneity despite using random effects models, likely due to the multitude of presentations. Hierarchical summary receiver operating characteristic models were performed for neutrophil-to-lymphocyte ratio and C-reactive protein and showed similar sensitivity at detecting mortality in ATAAD patients for each fixed specificity. Data showed that deranged inflammatory markers are associated with poorer outcomes in ATAAD; however, none of these measures provide suitable prognostic markers alone. Continued development of multifactorial risk scores, including inflammatory markers and other factors, such as thrombotic measures, may enable clinically relevant prognostic tools and risk stratification.

## Introduction

Aortic dissection is a life-threatening condition in which the aortic wall separates, allowing blood to enter between the layers. Aortic dissections involving the ascending aorta are Stanford Type A dissections. If not quickly identified and treated, Type A aortic dissection can lead to death, with mortality estimated at 1 to 2% of patients dying per hour for the first 48 hours after a dissection event due to organ/tissue malperfusion or aortic rupture. 1 Aortic dissection is treated by surgically replacing the aorta at the origin of the tear, with the extent of the surgical repair varying depending on the clinical presentation. 2

There is growing evidence that routinely assessed systemic markers of inflammation at hospital admission, particularly white blood cell counts (WBC) and the various ratios and indices generated from these counts, may be associated with poorer clinical outcomes. 3 4 5 If a specific biomarker can be identified as prognostically useful, this would provide clinicians with valuable information when performing a bedside risk assessment and, in turn, inform clinical decision-making. A preliminary search of PubMed, the Cochrane Database of Systematic Reviews, Prospero, and the Open Science Framework was conducted. Similar systematic reviews were identified, including one that indicated that C-reactive protein (CRP) was associated with a significantly increased risk of hospital mortality 6 and another which concluded an elevated neutrophil-to-lymphocyte ratio (NLR) was associated with aortic disease and mortality, although this analysis was not limited to acute Type A aortic dissection (ATAAD). 7 However, these reviews were not felt to address our aim to compare the effectiveness of admission inflammatory markers in predicting adverse outcomes in postoperative ATAAD patients. Therefore, a new systematic review would enable a clearer understanding of current knowledge. The review protocol was designed using the CHARMS checklist as a template, was prospectively registered with PROSPERO, and reported following PRISMA (Preferred Reporting Items for Systematic Reviews and Meta-Analyses) guidelines. 8 9 10 The review was designed to be broad and scoping. However, due to the large volume of identified literature, the authors decided to limit detailed meta-analysis to facilitate publication. Therefore, only WBC counts, neutrophil and lymphocyte counts, NLR, and CRP have been included.

## Methods

### Literature Searching and Screening

The review was prepared a priori and registered with PROSPERO (registration number CRD42022366509). A modified PICOTS framework was used to define study eligibility criteria ( Supplementary Table S1 , available in the online version only). 11 The search strategy aimed to include all relevant original research published after 1990 in English-language sources. Preliminary searches were used to define search terms, and the strategy was slightly adapted to suit each database (PubMed, Ovid Medline, and Scopus).

**Fig. 1:**
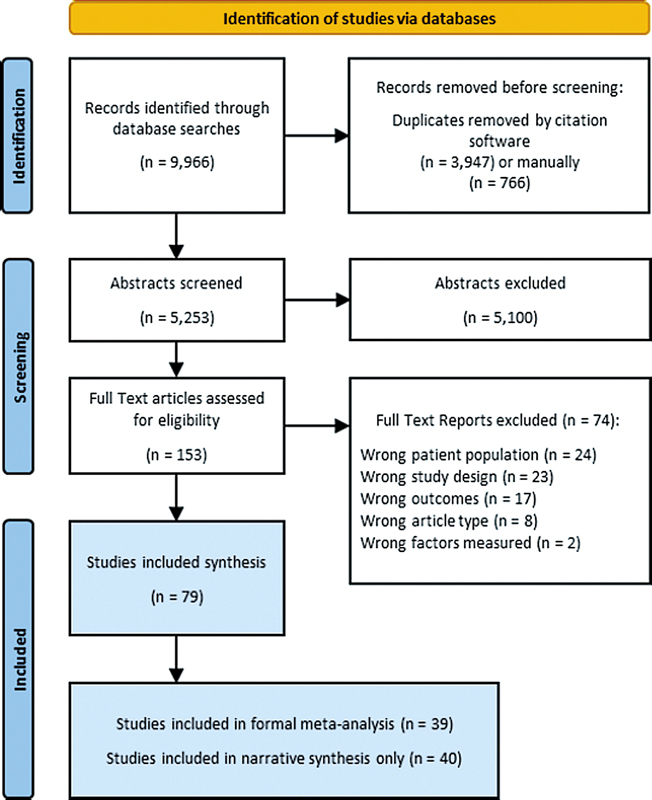
PRISMA flow diagram illustrating the searching and screening processes to identify studies for inclusion.

A total of 9,966 studies were identified, uploaded into JabRef 5.12, and duplicates removed. Abstract and full-text screening was performed against the inclusion–exclusion criteria, with reasons and numbers of studies excluded recorded and reported in a PRISMA flow diagram ( Fig. 1 ). Following duplicate removal and screening, 79 were considered eligible for study inclusion, with 39 included in the formal meta-analysis.

### Data Extraction and Synthesis

A custom-designed extraction form was produced within Extraction 2.0 in Covidence (Covidence.Org). Data extraction was conducted by a single reviewer (S.S. or A.M.). A second reviewer checked the data extraction for accuracy and consistency (S.S., A.M., or J.M.). Data extraction focused on citation identifiers, study design, study population, measured prognostic factors, and outcome measures including receiver operating characteristic (ROC) curve outputs and inflammatory marker values by clinical outcome measure.

A custom-designed quality assessment was also conducted within Extraction 2.0 in Covidence. This consisted of a modified quality in prognostic studies (QUIPS) tool ( Supplementary Table S2 , available in the online version only). Six domains were evaluated, including study participation, study attrition, prognostic factor measurement, outcome measurement, study confounding, and statistical analysis and reporting. Quality assessment formed part of the data extraction and was performed by a single reviewer (S.S. or A.M.), with a second reviewer assessing accuracy and consistency (S.S., A.M., or J.M.). Each study was evaluated using a traffic light system and rated as high (green), moderate (amber), or low (red) quality for each domain within the QUIPS tool. If a domain could not be adequately assessed, it was recorded as unknown or not applicable (gray) as appropriate.

### Synthesis and Meta-analysis

The original study proposal registered with PROSPERO aimed to assess various inflammatory markers and included different outcome measures. However, following data extraction, a pragmatic approach to meta-analysis, synthesis, and reporting was required. All identified studies were included in the preliminary study characteristics and summary tables. Next, studies comparing WBC count, neutrophil or lymphocyte count, NLR, and CRP levels by survival progressed to meta-analysis and synthesis. Studies comparing these markers for other postoperative outcomes were included in the narrative synthesis. Finally, extracted ROC data were included for meta-analysis if more than five studies evaluating the same outcome and measure were available.

#### Continuous Biomarker Analysis

Extracted data were divided by biomarkers of interest. Outcomes were grouped into mortality and morbidity measures. Meta-analysis was only performed if the number of studies included in each grouping was greater than 5. Meta-analysis was conducted using the ‘meta’ package in R (RStudio Version “Ocean Storm”). 12 Where available, the mean difference (MD) for each continuous biomarker was compared between survivors and nonsurvivors using a random effects model. The standardized mean difference method (SMD) was used to analyze CRP levels, as the scale of reported levels varied. Forest plots were used to visualize the results, including overall effect estimates and heterogeneity (assessed by the *I* ^2^ statistic). Data presented as median ± interquartile range (IQR) was transformed using the method described by Wan et al. 13 and the transformed means ± standard deviation (SD) were included within the meta-analysis. Extracted and transformed means were considered different subgroups within each analysis to facilitate clear interpretation.

#### Receiver Operator Characteristic Analysis

ROC curve variables were extracted for each included biomarker, including the area under the curve (AUC) with confidence intervals (CI), sensitivity, and specificity. If sufficient data (AUC, sensitivity, and specificity) were available from five or more studies, meta-analysis using a hierarchical summary receiver characteristic model (HSROC) was performed. To perform HSROC, the false positive, false negative, true positive, and false negative rates were calculated for each study based on the number of participants with and without the outcomes of interest and the reported sensitivity and specificity. HSROC was performed using R studio and STAN modelling following the method described by Banno et al. 14 The HSROC model estimated each marker's sensitivity to detect the outcome of interest for a range of specificities (0.55–0.95).

## Results

**Fig. 2:**
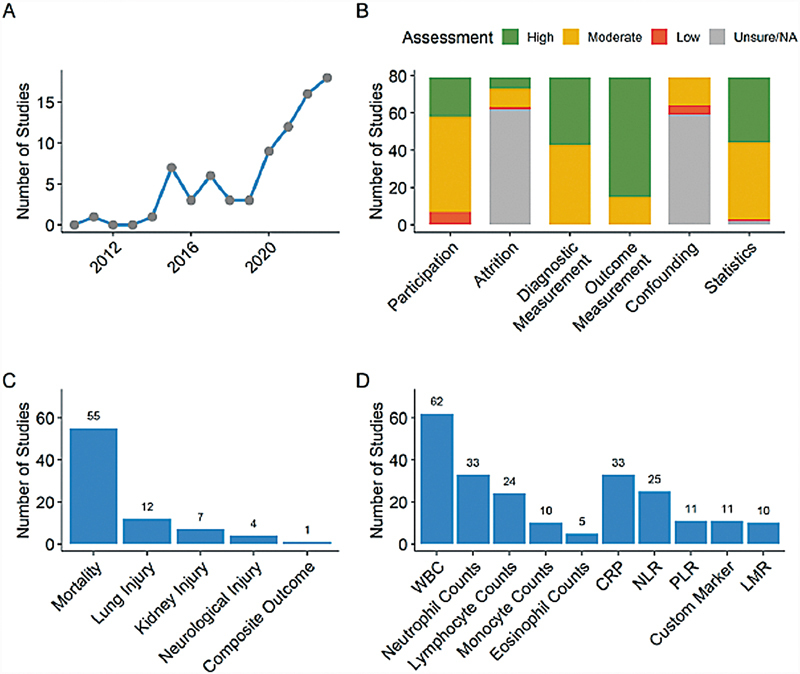
Study characteristics. ( **A** ) The number of articles included by publication year (2010–2023). ( **B** ) Overall results from quality assessments according to each QUIPS domain. ( **C** ) The frequency of primary outcomes from the included studies and ( **D** ) shows the frequency of each biomarker extracted in total from the included citations. White blood cell count (WBC), C-reactive protein (CRP), neutrophil-to-lymphocyte ratio (NLR), platelet-to-lymphocyte ratio (PLR), lymphocyte-to-monocyte ratio (LMR).

Publication date, prognostic markers, and primary outcome measures were characterized for each study. Despite searching all literature from 1990, very few publications were identified before 2015, with a notable increase in relevant articles from 2020 onward ( Fig. 2A ). This may correspond with growing interest in identifying biomarkers to aid clinical decision-making and the development of algorithms to stratify patients. Data extraction focused on the aims of this study; therefore, in some cases, only a portion of the available data was extracted and may not reflect the original aims of the included research. Similarly, the quality assessment scores evaluated studies based on their relevance to this systematic review ( Fig. 2B and Supplementary Table S3 , available in the online version only). A low or high score did not directly indicate a poor-quality or high-quality study; instead, it could indicate, for example, that the study's patient population was not limited to those relevant to this study's aims. Study participation was a key QUIPS domain, and while most studies focused on ATAAD patients, there was some heterogeneity. Included measures, particularly outcome measures, were generally clearly specified and highly reproducible. Given the nature of the research, the study attrition and confounding domains were frequently scored as either not applicable or unknown. Despite variation in methodological approaches and many studies being retrospective cohort analyses, most were still considered moderate or high quality.

Most studies (55/79) used mortality measures as their primary outcome measure ( Fig. 2C and Supplementary Table S3 , available in the online version only). The remainder of the studies had morbidity outcome measures, including postoperative lung injury (PLI) (12/79), acute kidney injury (AKI) (7/79), neurological injury (4/79), or a composite adverse outcome measure (1/79) ( Fig. 2C and Supplementary Table S4 , available in the online version only). Similarly, extracted biomarkers were predefined and might not include all markers discussed. All relevant biomarkers were extracted from each included study ( Fig. 2D ). Commonly extracted biomarkers were measures related to WBC count (62/79) or specific cell types, including neutrophil counts or percentages (33/79) and lymphocytes (24/79). Other less common cell counts included monocytes (10/79) and eosinophils (5/79). CRP was a common biomarker (33/79), and many studies used cell ratios, including NLR (25/79), platelet-to-lymphocyte ratio (PLR) (11/79), and lymphocyte-to-monocyte ratio (LMR) (10/79). Finally, several studies devised inflammatory ratios or indices that included several different markers of inflammation and coagulation or thrombosis (11/79). For brevity, detailed analysis was limited to WBC, neutrophil count, lymphocyte count, NLR, and CRP.

### White Blood Cell Counts

**Fig. 3:**
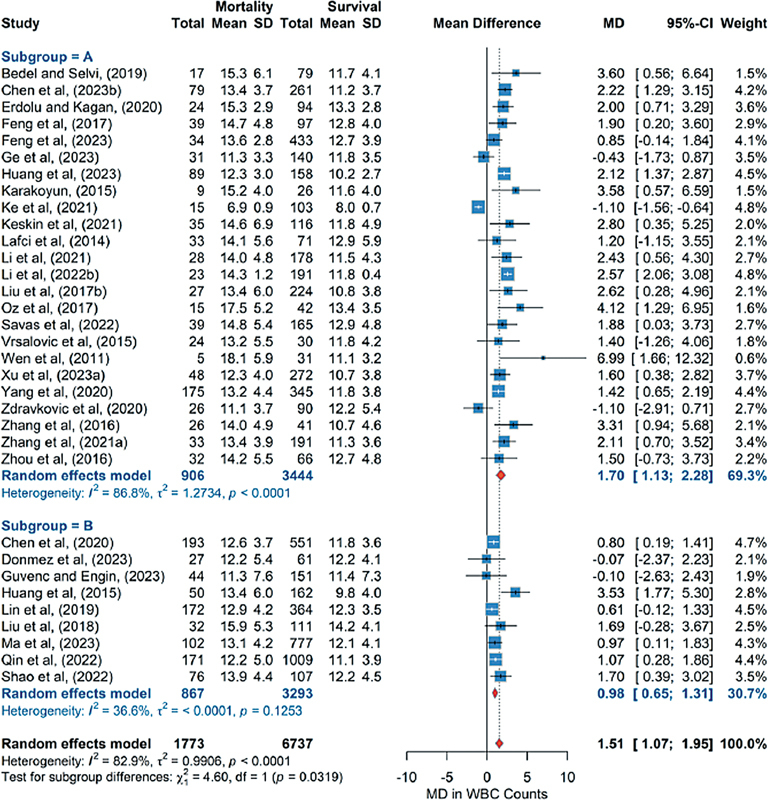
Forest plot showing meta-analysis of studies (Κ = 33) comparing white blood cell (WBC) counts in survivors and nonsurvivors following acute type A aortic dissection. Subgroup A includes studies that reported mean ± SD. Subgroup B includes studies that reported median (IQR). Individual study effect size is shown (blue squares). A random effects model estimate is indicated for each subgroup and an overall effect (red diamonds). IQR, interquartile range; SD, standard deviation.

A total of 33 studies, including a total of 8,510 observations, were included in a meta-analysis assessing the MD in baseline WBC count between ATAAD survivors and nonsurvivors 5 15 16 17 18 19 20 21 22 23 24 25 26 27 28 29 30 31 32 33 34 35 36 37 38 39 40 41 42 43 44 45 46 ( Fig. 3 ). Individual study effect sizes, represented by squares in the forest plot, indicate a range of MDs and varying degrees of precision as reflected by the CI size. Studies are subgrouped into those reporting mean ± SD (Subgroup A, Κ = 24) and studies reporting median and IQR (Subgroup B, Κ = 9). Reported median and IQR values were transformed using the method described by Wan et al. into estimated mean ± SD to facilitate inclusion. 13 Each subgroup was analyzed separately using a random effects model. While both models indicated that WBC counts were lower in survivors than in nonsurvivors, the MD in Subgroup A was greater than in Subgroup B (MD = 1.70 [CI = 1.13, 2.28] vs. MD = 0.98 [CI = 0.65, 1.31], *p* = 0.03). The overall pooled effect using a random effects model resulted in a statistically significant MD of 1.51 [CI = 1.07, 1.95], indicating that, on average, WBC counts were higher in mortality cases than in survivors (Z = 6.71, *p* < 0.0001). The *I* ^2^ statistic of 83% suggests substantial heterogeneity across the included studies. This level of heterogeneity indicates a significant variation in the effect sizes that may be due to differences in study populations, methods, or other factors.

Additional studies were identified containing results relating to baseline WBC counts that were not included within the primary meta-analysis. Four studies divided patients into cohorts based on baseline WBC counts (low or high) and compared mortality outcomes. Of these two showed that mortality cases in the low WBC (<11) groups were lower than in the high (>11) WBC count group for 30-day mortality (14/340 [4%] vs. 47/216 [22%] 47 and in-hospital mortality (12/149 [8%] vs. 38/182 [21%] 48 ). Similarly, another study showed that a mixed cohort of patients with hight WBC ( > 15) experienced 34.64& (53/153) in-hospital mortality. 49 However, Suzuki et al. found no difference in either 30-day mortality (WBC < 11 21/211 [10%] vs. WBC >11 14/255 [5%]) or in-hospital mortality (WBC < 11 23/211 [11%] vs. WBC > 11 19/255 [7%]). 50

Other studies considered postoperative morbidity outcomes, including renal, 51 52 53 54 neurological, 55 56 57 58 and respiratory dysfunction 59 60 61 62 63 64 65 66 67 68 69 ( Supplementary Table S5 , available in the online version only) or a composite adverse outcome. 70 These studies used various outcome measures, making direct comparisons more difficult. For example, studies grouped into PLI included measures such as prolonged mechanical ventilation, 59 62 tracheostomy, 65 or hypoxemia. 60 63 64 69 Nevertheless, 10 of the included 11 studies showed higher WBC counts were associated with PLI. 59 60 61 63 64 65 66 67 68 69 Similarly, elevated WBC counts were associated with poorer clinical outcomes related to renal function in all studies 51 53 71 except one, 52 and a further three studies showed an association between higher WBC counts and neurological injury. 55 56 57 Only two studies did not show a significant association between higher WBC and prolonged mechanical ventilation 62 or transient neurological dysfunctions. 56 Finally, Tang et al. used a composite in-hospital adverse outcome measure, which included in-hospital mortality, Stage 3 AKI or stroke but did not demonstrate a statistically significant difference in baseline WBC counts between ATAAD patients with or without adverse outcomes. 70

### Neutrophil Counts

**Fig. 4:**
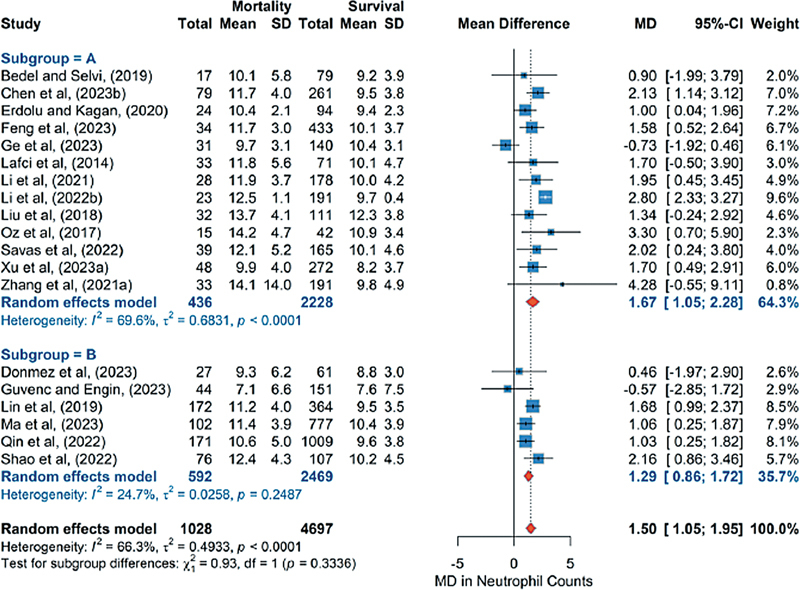
Forest plot showing meta-analysis of studies (Κ = 19) comparing neutrophil counts in survivors and nonsurvivors following acute type A aortic dissection. Subgroup A includes studies that reported mean ± SD, Subgroup B includes studies that reported median (IQR). Individual study effect size is shown (blue squares). A random effects model estimate is indicated for each subgroup and an overall effect (red diamonds). IQR, interquartile range; SD, standard deviation.

A total of 5,725 observations made across 19 studies were included in a meta-analysis assessing the MD in admission or baseline neutrophil count between ATAAD survivors and nonsurvivors 5 15 16 18 19 24 25 26 28 29 32 36 39 40 42 43 44 45 46 ( Fig. 4 ). Subgroup A (Κ = 13) contains studies reporting mean ± SD, whereas Subgroup B (Κ = 6) includes transformed median and IQR values. The model produced for each subgroup was similar ( *p* = 0.33), and the overall pooled effect resulted in a statistically significant MD of 1.50 [CI = 1.05, 1.95], indicating that, on average, neutrophil counts were higher in mortality cases than in survivors (Z = 1.50, *p* < 0.0001). The *I* ^2^ statistic of 66% suggests substantial heterogeneity across the included studies. Other studies considered neutrophil counts in the context of postoperative morbidity rather than mortality, including renal, 52 54 respiratory, 60 61 67 72 and neurological dysfunction. 58 High baseline neutrophil percentage or count was significantly associated with postoperative AKI 54 including Stage 3 AKI. 52 Similarly, several studies found elevated neutrophil counts or ratios were associated with postoperative hypoxaemia, 60 pneumonia, 61 and postoperative oxygenation impairment. 67 While Wang et al. found that patients divided into low (<0.824) or high (>0.824) baseline neutrophil percentages had different rates of acute lung injury (neutrophil ratio < 0.824 51/251 [20%] vs. neutrophil ratio > 0.824 81/228 [36%]). 72 However, Lin et al. did not find a significant association between elevated neutrophil counts and postoperative delirium. 58

### Lymphocyte Counts

**Fig. 5:**
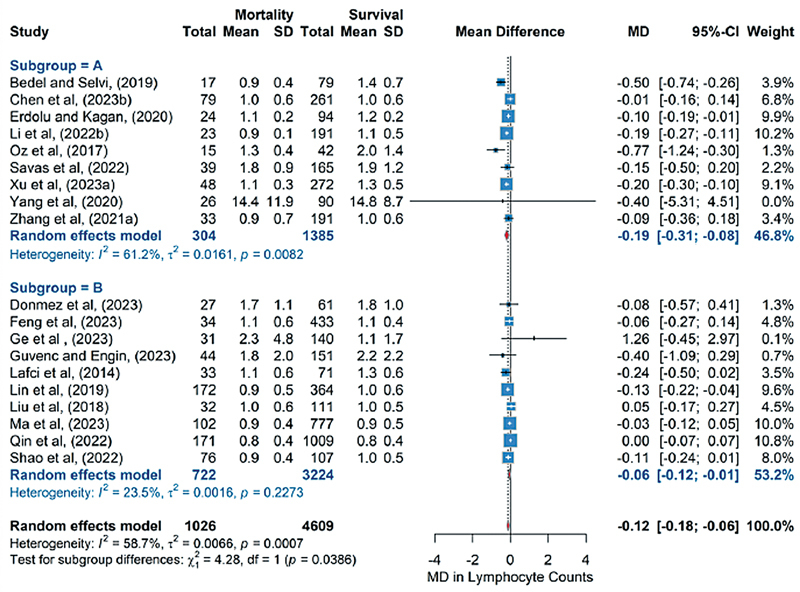
Forest plot showing meta-analysis of studies (Κ = 18) comparing lymphocyte counts in survivors and nonsurvivors following acute type A aortic dissection. Subgroup A includes studies that reported mean ± SD. Subgroup B includes studies that reported median (IQR). Individual study effect size is shown (blue squares). The random effects model estimate is indicated for each subgroup and an overall effect (red diamonds). IQR, interquartile range; SD, standard deviation.

A total of 5,519 observations made across 18 studies were included in a meta-analysis assessing the MD in admission or baseline lymphocyte counts between ATAAD survivors and nonsurvivors 5 15 16 18 19 24 26 28 29 32 36 39 40 42 43 44 45 46 ( Fig. 5 ). Subgroup A (Κ = 8) contains studies reporting mean ± SD, whereas Subgroup B (Κ = 10) includes transformed median and IQR values. In contrast to other inflammatory markers, both models indicated that lymphocyte counts were higher in survivors than nonsurvivors. The MD in Subgroup A was greater than Subgroup B (MD = −0.19 [CI = −0.31, −0.08] vs. MD = −0.06 [−0.12, −0.01], *p* = 0.04). The overall pooled effect resulted in a statistically significant MD of −0.12 [CI = −0.18, −0.06], indicating that, on average, lymphocyte counts were lower in mortality cases than in survivors (Z = −4.11, *p* < 0.0001). The *I* ^2^ statistic of 61% suggests substantial heterogeneity across the included studies. Only one additional study reported lymphocyte levels comparing survivors and nonsurvivors. Yu et al. found that lymphocyte percentage was lower in nonsurvivors than survivors, potentially reflecting an increase in total WBC count and a decrease in lymphocytes. 73 Studies evaluating morbidity outcomes also found lower lymphocyte counts were associated with AKI 54 but not with delirium 58 or postoperative oxygenation impairment. 67

### Neutrophil-to-Lymphocyte Ratio

**Fig. 6:**
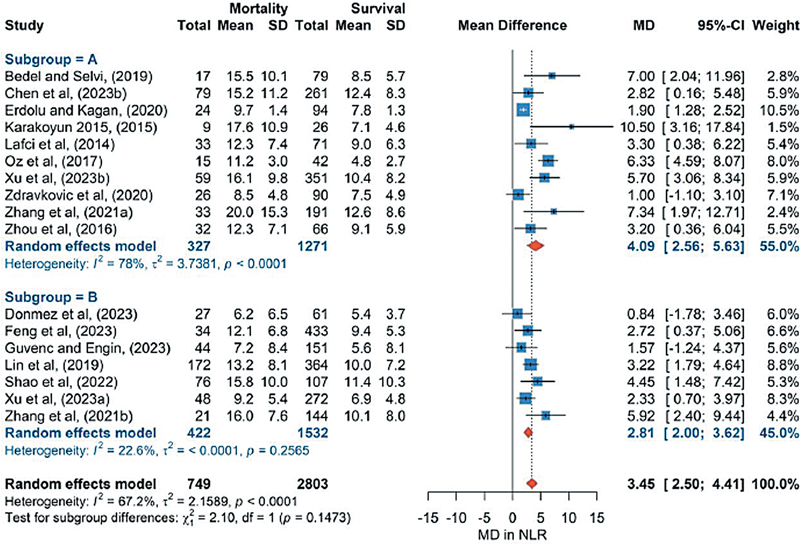
Forest plot showing meta-analysis of studies (Κ = 17) comparing the neutrophil-to-lymphocyte ratio (NLR) in survivors and nonsurvivors following acute type A aortic dissection. Subgroup A includes studies that reported mean ± SD. Subgroup B includes studies that reported median (IQR). Individual study effect size is shown (blue squares). The random effects model estimate is indicated for each subgroup and an overall effect (red diamonds). IQR, interquartile range; SD, standard deviation.

Seventeen studies, totaling 3,552 observations, were included in the meta-analysis assessing the MD in admission or baseline NLR between ATAAD survivors and nonsurvivors 5 15 16 18 21 24 28 32 34 36 37 39 40 42 46 74 75 ( Fig. 6 ). Subgroup A (Κ = 10) contains studies reporting mean ± SD, and Subgroup B (Κ  = 7) includes transformed median and IQR values. The model produced for each subgroup was similar ( *p* = 0.15), and the overall pooled effect resulted in a statistically significant MD of 3.45 [CI = 2.50, 4.41], indicating that, on average, the NLR was higher in mortality cases than in survivors (Z = 7.10, *p* < 0.0001). The I ^2^ statistic of 67% suggests substantial heterogeneity across the included studies. One further study divided ATAAD patients into two cohorts based on their baseline NLR (low <6.0 or high >6.0) and found that in-hospital mortality was lower in the low NLR group (NLR < 6.0, 10/93 [11%]) than in the high NLR group (NLR > 6.0, 38/91 [31%]). 4 Another found that the risk of mortality was highest in ATAAD patients with an NLR between 7 and 20. 76 Likewise, elevated baseline NLR was associated with an increased risk of postoperative oxygenation impairment 67 but not with a composite measure of in-hospital adverse outcomes 70 or acute renal failure. 77

### C-Reactive Protein

**Fig. 7:**
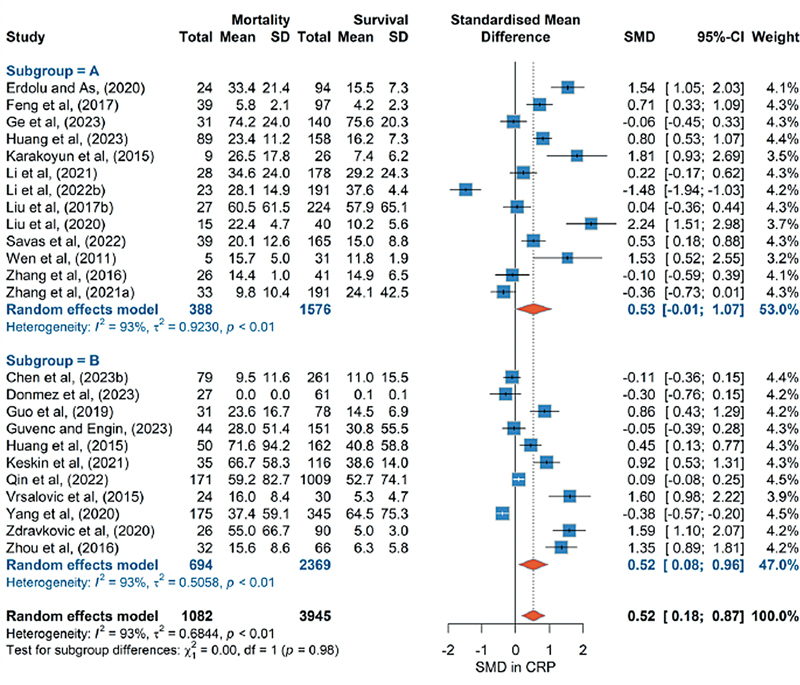
Forest plot showing meta-analysis of studies (Κ = 24) comparing baseline CRP levels in survivors and nonsurvivors following acute Type A aortic dissection. Subgroup A includes studies that reported mean ± SD. Subgroup B includes studies that reported median (IQR). Individual study effect size is shown (blue squares). The random effects model estimate is indicated for each subgroup and an overall effect (red diamonds). IQR, interquartile range; SD, standard deviation.

A total of 5,027 observations made across 24 studies were included in a meta-analysis assessing the SMD in admission or baseline CRP levels between ATAAD survivors and nonsurvivors 15 16 17 19 20 21 23 25 26 27 29 30 31 33 34 35 36 37 39 40 41 45 78 79 ( Fig. 7 ). SMD was used to reduce the impact of different reporting scales and measurements. Subgroup A (Κ = 13) contains studies reporting mean ± SD, whereas Subgroup B (Κ = 11) includes transformed median and IQR values. There was no statistically significant difference between subgroups A and B ( *p* = 0.9764), and the overall pooled effect was an SMD of 0.5227 [CI = 0.1781, 0.8672], indicating that CRP was lower in survivors than nonsurvivors (Z = 2.97, *p* = 0.003). The *I* ^2^ statistic of 93% suggests very substantial heterogeneity across the included studies. Several studies also compared CRP levels by postoperative morbidity outcomes. Wu et al. found baseline CRP was significantly higher in patients who had acute lung injury compared with those who did not (32.27 ± 0.49 vs. 60.38 ± 19.15, *p* < 0.1). 66 Similarly, Tang et al. showed that patients experiencing in-hospital adverse outcomes had higher baseline CRP than those who did not (7.45 [5.0–20.6] vs. 9.2 [5.85–55.0], *p* = 0.029). 70 However, other studies did not find a significant difference in baseline CRP between patients with prolonged mechanical ventilation, 59 62 hypoxaemia, 60 AKI, 53 or transient neurological deficit. 56

### Other Inflammatory Markers

Other inflammatory markers identified in the literature include monocyte and eosinophil counts and several ratios and indices such as PLR, LMR, and custom-designed inflammatory markers. A formal meta-analysis of these markers was not conducted. A total of five studies considered baseline eosinophil counts or percentages. Some showed significant differences between survivors and nonsurvivors, 45 46 whereas others failed to detect a significantly different eosinophil count in either mortality 34 44 or postoperative AKI. 54 Ma et al. found significantly higher admission monocytes in mortality cases compared to survivors, in contrast to several studies that did not show a significant difference. 15 29 42 43 44 45 46 In contrast, higher baseline monocytes were associated with postoperative AKI 71 and oxygenation impairment 67 in ATAAD patients but not with postoperative delirium. 58 Several studies considered monocytes as part of a ratio, notably the LMR. They found that baseline LMR values were significantly lower in ATAAD patients who did not survive, 15 42 44 46 experienced a composite adverse event outcome, 70 had AKI, 71 and impaired postoperative oxygenation. 67 Furthermore, Ma et al. compared in-hospital mortality and AKI by LMR level and found that patients with low LMR (<1.51) were more likely to have poor outcomes, including in-hospital mortality and AKI. 80

Another commonly used ratio was the PLR. This marker includes a thrombotic measure, the platelet count but has been included as several studies referenced this measure. Bedel and Selvi found ATAAD patients who did not survive had a significantly higher PLR than survivors, but other included studies did not find a significant difference in PLR for mortality measures 5 15 32 39 46 or morbidity outcomes. 67 77 A potential explanation for the inconclusive findings comes from Xie et al., who identified a U-shaped relationship between PLR and ATAAD postoperative mortality. 81 Finally, several studies used custom ratios or developed indices or algorithm-based inflammatory scores. 32 74 82 83 84 85 86 87 88 89 Many of these scores combine systemic inflammatory and thrombotic or coagulation markers to generate a prognostic risk score to identify ATAAD patients at risk of mortality. Although the exact combination of markers included in these scores varies between studies, most include a combination of platelet and inflammatory cell count measures.

### Receiver Operating Characteristic Analysis

ROC models are commonly used to assess a marker's sensitivity and specificity in detecting an outcome of interest in a population. For effective meta-analysis of these data, the number of true positive, false positive, true negative, and false negative patients, based upon a marker cutoff value, is required. However, these data are not generally reported in the literature; instead, reported data include AUC with CIs, plus the sensitivity and specificity in detecting the outcome of interest. Of the included 79 studies, 30 reported ROC analysis data 4 5 16 17 20 21 24 25 28 30 34 38 40 42 48 51 70 71 74 79 82 83 84 86 87 88 90 91 ( Supplementary Table S6 , available in the online version only). Many of these studies evaluated a marker's ability to discriminate mortality outcomes; however, morbidity outcomes were also assessed. The most commonly assessed markers were CRP and NLR, but many studies also assessed custom inflammatory markers and algorithms that contained inflammatory elements ( Supplementary Table S6 , available in the online version only).

**Fig. 8:**
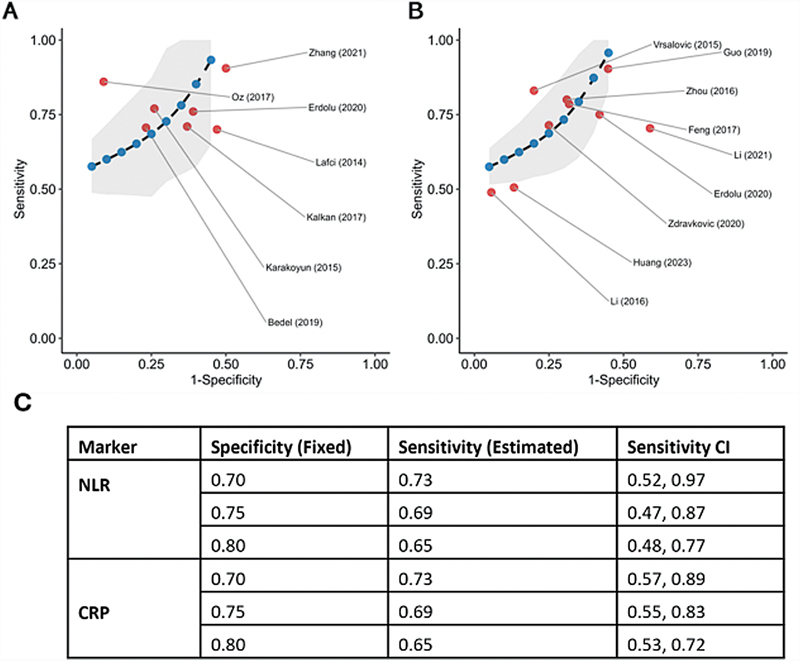
HSROC plot showing summary estimates of sensitivity plotted against specificity (blue dots) with the confidence interval shaded in grey for ( **A** ) neutrophil-to-lymphocyte ratio (NLR) and ( **B** ) C-reactive protein (CRP) as a prognostic marker in acute Type A aortic dissection (ATAAD). The sensitivity and specificity of individual studies are plotted. Each included study is labelled and marked in red. ( **C** ) HSROC modelled point estimates marker sensitivity in detecting mortality for fixed specificities. HSROC, hierarchical summary receiver characteristic.

Unfortunately, in many cases, insufficient data were available to facilitate meta-analysis. Meta-analysis using a HSROC model was performed if more than five studies presented complete data (AUC, sensitivity, and specificity) for a specific marker of interest. This resulted in HSROC models being produced for NLR (Κ = 7) ( Fig. 8A ) and CRP (Κ = 9) ( Fig. 8B ). Each marker's sensitivity was modelled against fixed specificity to obtain estimate values based on the included studies ( Fig. 8C ). Results indicated that NLR and CRP were similarly sensitive in detecting mortality in ATAAD patients. However, the CIs for CRP were slightly narrower at each sensitivity point estimate.

Five studies evaluated WBC counts prognostic value in determining mortality outcomes. Although the outcomes of interest did vary, the reported AUC values were comparable across studies, with Li et al. reporting an AUC = 0.637 for mortality, 82 Li et al. reporting an AUC = 0.641 (CI = 0.567, 0.71) for in-hospital mortality, 25 Liu et al. reporting an AUC = 0.578 (CI = 0.537, 0.62) for 90-day mortality, 84 Liu et al. reporting an AUC = 0.628 (CI = 0.584, 0.673) for 30-day mortality. 86 Similarly, three studies included ROC analysis of neutrophil counts for in-hospital mortality 25 87 and 30-day mortality outcomes. 86 The reported AUC values were 0.653 (CI = 0.58, 0.721), 25 0.568 (CI = 5.21, 0.614), 86 and 0.598 (CI = 0.563, 0.634). 87 Only Liu et al. performed ROC analysis of lymphocyte counts for 30-day mortality (AUC = 0.511 [CI = 0.468, 0.554]). 86

## Discussion

This review was undertaken using established and systematic methods. The low incidence and extreme emergent nature of ATAAD have resulted in most investigations in this setting being retrospective and observational. Meta-analysis techniques have enabled the pooling of observations from different studies and demonstrate agreement that inflammatory markers could have prognostic value; however, the effect size varies considerably. The increase in WBC and neutrophil counts in nonsurvivors compared with survivors is small but statistically significant. In contrast, lymphocyte counts show the opposite effect, with nonsurvivors having lower counts. Combining the impact of lower lymphocytes and higher neutrophils into a single marker, NLR, leads to a more pronounced effect, making differences easier to distinguish, which could help determine cutoff values if used clinically. This has led to considerable interest in using NLR as a prognostic marker across many clinical conditions, including cancer, COVID and cardiovascular disease. 92 93 94 A systematic review by Xu et al. identified elevated NLR was associated with poor prognosis in aortic disease patients, including those with aneurysms and dissection. 7 Similarly, we identified elevated CRP was associated with a poorer outcome in ATAAD patients. Hsieh et al. performed an excellent meta-analysis to assess the effectiveness of CRP as a prognostic marker of in-hospital mortality in ATAAD patients. 6 They identified that elevated CRP level was associated with a significantly increased risk of in-hospital mortality. 6

Although there is considerable agreement within the literature, a key question for clinicians is whether these easily measured surrogate markers can inform clinical decision-making, facilitating a move toward algorithm-driven care. The possibility of differentiating which patients should undergo open surgery, determining the extent of surgical repair undertaken, or determining in which patients additional intraoperative or postoperative management strategies to reduce the inflammatory response should be employed. ROC analysis can determine cutoff values to dichotomize patients into high-risk and low-risk groups. However, for a marker to be useful, it must be sufficiently sensitive and specific in detecting the outcome of interest. The HSROC meta-analysis of NLR and CRP yielded very similar results. Both markers showed good sensitivity at high specificities; however, neither marker could be reliably used to distinguish between patients with different outcomes, limiting their utility in algorithm-based risk assessments. Using a bivariate model, Hsieh et al. reported a pooled sensitivity of 0.77 (CI = 0.69–0.84) and specificity of 0.72 (CI = 0.67–0.78) of CRP to predict in-hospital mortality. 6 This alternative meta-analysis method indicated slightly better performance than our HSROC modelling. At a fixed specificity of 0.75 for both NLR and CRP, sensitivity was predicted at 0.69 (NLR [CI = 0.47, 0.87] and CRP [CI = 0.55, 0.83]), although results were in the CI range. A quick effectiveness test can be performed by summing sensitivity and specificity. A value of two is perfect but 1.5 a useful threshold value. 95 A sum of 1.44 for our HSROC and 1.49 for Hsieh et al. both fall just under this threshold, indicating insufficient clinical value. Integrating inflammatory markers with other clinical factors may enhance decision-making. Several included studies demonstrated that custom-designed composite measures of inflammation and thrombosis or coagulation were potentially more effective. 32 74 82 83 84 85 86 87 88 89

Despite the lack of certainty regarding the development of prognostic markers, these findings show that ATAAD patients experiencing a greater inflammatory response at admission are at greater risk of poor outcomes during their postoperative period. ATAAD is a complex disorder with many factors at play. The clinical urgency of ATAAD is well understood, with delayed diagnosis increasing the risk of death due to rupture or complication. However, the association between delayed diagnosis and inflammation is not fully elucidated. The inflammatory response is complex and evolves with time. The neutrophil response is rapid, with neutrophils released from the bone marrow within minutes, while increasing CRP levels may occur more slowly over several hours. Therefore, we hypothesized that differing neutrophil counts or NLR between patients may reflect a difference in the time from the index event to the test performed rather than a different response. Few studies provided time from the index event, and none stratified patients by time. Six studies compared time from index event with inflammatory marker levels with no significant differences reported. 4 45 47 85 89 90 Considering the impact of time from the ATAAD event in future research may provide more granular detail, potentially improving the predictive power of any inflammatory markers.

Inflammatory markers investigated in this review could be affected by presence of additional factors associated with the acute phase response, including tamponade, malperfusion, and ischemia. Many studies didn't report incidence of these factors with some actively excluding them. Percentage of incidents of cardiac tamponade was not significantly different between high and low NLR groups 4 or when stratifying by systemic coagulation-inflammation index (SCI). 84 However, Li et al. 82 found patients with higher simplified thrombo-inflammatory prognostic score (sTIPS) were more likely to suffer pericardial tamponade (2.4% of sTIPS0, 16.7% of sTIPS1, 23.5% of sTIPS2; *p* = 0.038).

Two studies report no association of malperfusion with inflammatory profile 89 or PLR tertiles. 81 Coronary and cerebral malperfusion were included within the 12 variables contributing to the inflammatory predictive model developed by Liu et al. 86 The same group also reported preoperative malperfusion as significantly negatively correlated with SCI index ( *p* < 0.001), with high SCI associated with improved survival (malperfusion incidence rates of 30.9% in high SCI, 35.7% in mid SCI, and 42.9% in low SCI). 84

Heart ischemia was not associated with inflammatory profile in two reported studies. 85 89 Qin et al. 45 divided patients into low and high circulating EOS eosinophil count on admission and reported more patients in the lower EOS group developed cerebral ischemia attack (10.0 vs. 5.8%, *p* = 0.010) than the higher EOS group, with the lower EOS count group associated with increased mortality. Suzuki et al. 50 also divided their cohort into high and low WBC and reported mesenteric ischemia as more prevalent in the elevated WBC group than in the normal WBC count group ( *p* = 0.028), whereas myocardial ischemia was not significantly different. Interestingly, an absence of coronary ischemia was found to be a factor related to high WBC ( *p* = 0.027). However, the authors note that this is the first study suggesting this, and it may be related to relatively small patient numbers. 50 Li et al. developed a predictive model with Systemic Immune-Inflammation (SII) index and additional clinical factors, including preoperative limb ischemia as a variable. 83 Patients in the low SII group had higher proportions of cerebral ischemia (9.88 vs. 3.30%, *p* = 0.044), whereas patients in the high SII group had increased postoperative bowel ischemia (5.49 vs. 0.99%, *p* = 0.004). Li et al. described in-hospital complications of acute and myocardial ischemia to be associated with increasing sTIPS; acute limb ischemia, sTIPS0 2.4%, sTIPS1 20.8%, sTIPS2 5.9% ( *p* = 0.018), myocardial ischemia, sTIPS0 14.6%, sTIPS1 8.3%, sTIPS2 35.3% ( *p* = 0.028). 82

An additional factor that may influence the inflammatory response is the extent of dissection. Similarly to factors explored above many studies didn't define or compare dissection extent. As expected, those that reported a higher inflammatory response was associated with increased extent of dissection. Liu et al. included DeBakey classification and extent of dissection within SCI index, with high SCI index associated with increased 90-day survival. Extension of the dissection to include arch or descending was associated with low SCI ( *p* < 0.001). 84 Extent of dissection extension (ascending only, extending to arch or descending) was also included within the 12-parameter inflammatory based prediction model for mortality developed by Liu et al. 86 Ma et al. also showed involvement of the descending aorta (but not the arch) was associated with low LMR ( *p* = 0.04), with low LMR associated with higher incidences of worse postoperative outcomes including in-hospital mortality and AKI. 80 More detailed analysis of branch involvement by Li et al. identified coronary artery involvement to be significantly associated with thromboinflammatory score (sTIPS0 0.0%, sTIPS1 2.1%, sTIPS3 17.6% [ *p* = 004]), whereas other arteries (renal, common iliac, internal or external iliac, mesenteric or arterial branches of aortic arch) involvement were not associated. 82 Suzuki et al. showed distal extent of dissection was significantly associated with WBC count ( *p* < 0.001), with extension to the iliac artery significantly more prevalent in the elevated WBC count group ( *p* = 0.006). 50 Authors suggest that dissection morphology influences the acute phase systemic inflammatory response associated with an elevated WBC count.

Regardless of whether inflammatory markers are employed clinically to stratify ATAAD patients, the inflammatory component of ATAAD should be considered. Targeted treatments to reduce inflammation could improve patient outcomes, such as steroid therapies, cytokine adsorption during surgery and the immediate postoperative period, or other anti-inflammatory therapeutics. Despite evidence for inflammatory markers predicting postoperative morbidity being more mixed, there were still numerous studies linking inflammatory markers with postoperative morbidity in ATAAD patients. Reducing the size of the inflammatory response is likely to improve postoperative recovery and reduce the frequency or severity of postoperative morbidity, including AKI and lung injury.

One of the studies included within this review investigated the effect of anti-inflammatory pharmacotherapy. 85 They identified that ulinastatin treatment reduced ventilator time from 54 to 41 hours ( *p* = 0.048) in patients subphenotyped as hyperinflammatory. Ulinastatin acts by suppressing proinflammatory cytokines and upregulating the release of anti-inflammatory mediators, including suppression of neutrophil activity. 96

Cytokine filters or hemoadsorption (HA) cartridges are used during a range of surgical procedures, particularly cardiac surgery aimed to reduce excessive inflammatory burden. They have been shown to improve postoperative patient outcomes, including mortality and ICU stay during emergency surgery and those associated with high inflammatory responses e.g., infective endocarditis. 97 Use of hemoadsorption therapy in these patient groups showed a significant reduction in CRP at 1 day following surgery compared with comparable surgery without HA use. However, WBC and other inflammatory markers did not show a difference. Wang et al. investigated use of HA cartridges within ATAAD surgery. 98 They observed a lower incidence of postoperative AKI (25.4 vs. 44.6%, *p* = 0.032) and severe acute respiratory distress syndrome (18.3 vs. 35.1%, *p* = 0.040) in the HA group, associated with a reduced increase in serum IL-6 levels.

Baseline inflammatory markers likely possess the ability to predict which patients would benefit from these approaches to reduce excess inflammatory response, but additional studies and data are needed before they would be ready for clinical implementation. Several studies included within this review proposed inclusion of inflammatory markers along with additional clinical factors likely associated with modifying or contributing to the inflammatory response to predict postoperative outcomes. Collection and reporting of additional data on potential contributing factors such as time from index event, extent of dissection, and presence of malperfusion, ischemia, or tamponade would enable more detailed analysis of the effects of these on modulating the inflammatory response and improve its potential predictive capabilities and incorporation into useable models.

### Limitations

Although random effects models were used, significant heterogeneity was observed, which is unsurprising given the complexity of ATAAD and the varied methodological research approaches. Not all studies contained a single population, with some including chronic or Type B dissections. Additional factors contributing to study heterogeneity could include the variation in ATAAD management across health care systems. Differences in models of care can lead to disparities in access to treatment. Factors such as geography, referral pathways, and availability of advanced surgical options can vary widely, in addition to clinical elements influencing overall inflammation levels that were not considered, including the extent of the aortic dissection, the patency of the false lumen, and the presence of organ ischemia due to impaired blood flow. Yet, despite this heterogeneity, the meta-analyses strongly suggest a correlation between inflammation markers and poorer postoperative outcomes.

## Conclusion

ATAAD is a traumatic and life-threatening condition associated with high levels of inflammation. Deranged inflammatory markers measured at admission have been associated with poorer outcomes. Despite these findings, HSROC analysis indicated that while NLR and CRP showed good sensitivity and specificity, these markers alone may not be sufficient to be useful as prognostic markers. Further investigation into the nature of the inflammatory response in ATAAD could provide opportunities to improve patient management by developing prognostic biomarkers and targeted treatment options to support patients through their operative journey.
