# Supplementary material for: Systematic Review and Meta-analysis of Admission Inflammatory Biomarkers for Evaluating Prognosis in Acute Type A Aortic Dissection
Source: Aorta (Stamford). 2025 Oct 7;13(03-04):79–93. doi: 10.1055/a-2693-4070 (PMC13300749; doi:10.1055/a-2693-4070)
Supplement: Supplementary file 1 — Supplementary Material [file 10-1055-a-2693-4070-s250005.pdf]

Thieme

Supplementary Materials

Supplementary Table 1. Inclusion-Exclusion Criteria defined by modified PICOTS framework.

	Inclusion	Exclusion
**Population**	Acute Type A aortic dissection including acute aortic syndromes such as intramural haematoma and penetrating aortic ulcer and mixed patient cohorts	Type B aortic dissection<br>Chronic dissection (Type A or Type B)<br>Aortic aneurysm
	Adult	Paediatric
**Index prognostic factor**	Not applicable as there is no routine clinical prognostic factor in use.	
**Comparator prognostic factors**	Systemic markers of inflammation that are routinely measured at admission including:<br>• Total white blood cell count<br>• White cell fractions as ratios or counts<br>• C-reactive protein<br>• Scores or indices generated using routinely collected counts, fractions or factors	Biomarkers that are not routinely measured, for example, cytokine or matrix metalloproteinase levels
**Outcome**	Receiver operator characteristic (ROC) analysis results related to postoperative mortality and morbidity.<br>Comparative biomarker levels between different prognostic cohorts	Narrative findings without clear statistical analysis in the form of ROC, univariate and/or multivariate analysis.<br>Results that do not relate to postoperative mortality or morbidity outcomes
**Timing**	Biomarker sampling must be at admission or prior to surgical intervention.	Biomarker sampling during or after surgical repair.<br>Outcome data must be following surgical repair.
**Source Type**	Original research published in an English language peer-reviewed journal since 1990.	Conference abstracts, review articles, opinion pieces and editorials.<br>Original research published before 1990 or in a language other than English

Supplementary Table 2. Quality in prognostic studies (QUIPS) tool for quality assessment of included studies scoring criteria.

Number	Domains	Items for consideration
**1**	Study participation	Adequate participation by eligible persons<br>Clearly defined clinical groups e.g. ATAAD only, mixed dissection cohort<br>Description of baseline characteristics<br>Adequate description of inclusion and exclusion criteria
**2**	Study attrition	Adequate number of study participants have complete data<br>Reasons for loss to follow-up are provided<br>There are no important differences between those who have complete data and those who do not
**3**	Prognostic factor measurement	A clear definition of the prognostic factor is given<br>Method of prognostic factor is valid and reliable<br>Continuous variables are reported or appropriate cut-points are used<br>The method of measurement is the same for all study participants<br>An adequate number of the study sample has complete data<br>Appropriate methods are used for missing data
**4**	Outcome measurement	A clear definition of the outcome is provided<br>Method of outcome measurement used is adequately reliable and valid<br>The method and setting outcome measurement is the same for all study participants
**5**	Study confounding	All important confounders are measured<br>Clear definitions of confounders measured are provided<br>The method and setting of confounding measurement<br>Important confounders are accounted for in the analysis
**6**	Statistical analysis and reporting	Sufficient presentation of data to assess the adequacy of the analysis<br>Strategy for model building is appropriate and is based on a conceptual framework or model<br>The selected statistical model is adequate for the design of the included study<br>There is no selective reporting of results

Supplementary Table 3. Summary of study characteristics for studies using mortality measures as a primary outcome measure.

Author	Year	Study Population	Operated (%)	Sample Size	Mean Age	Outcome of Interest	Extracted Biomarkers	QUIPS 1 2 3 4 5 6
Bedel and Selvi[5]	2019	ATAAD	89.6	96	63.7	In-hospital Mortality	WBC, NC, LC, NLR, PLR	🟡⚪🟢🟢⚪🟡
Chen *et al.*[38]	2020	ATAAD	87.4	744	Survived=51.8, Deceased=58.5	30-day Mortality	WBC, LMR, LNR	🟢🟢🟡🟢🟡🟡
Chen *et al.*[15]	2023(b)	ATAAD	100	340	53.32	In-hospital Mortality	WBC, NC, NLR, PLR, LMR, CRP, MC	🟢⚪🟢🟢⚪🟢
Dönmez *et al.*[39]	2023	Mixed Cohort	N/A	88	N/A	30-day Mortality	WBC, NC, LC, NLR, PLR, CRP	🔴⚪🟡🟢⚪🟢
Erdolu and Kagan[16]	2020	ATAAD	100	118	57	In-hospital Mortality	WBC, NC, LC, NLR, CRP	🟡⚪🟢🟢⚪🟢
Fan *et al.*[47]	2015	ATAAD	70	570	46	Mortality	WBC	🟡⚪🟡🟢🟡🟢
Feng *et al.*[17]	2017	ATAAD	72	136	53.7	Mortality	WBC, CRP	🟡🔴🟢🟢⚪🟡
Feng *et al.*[18]	2023	ATAAD	100	467	52.06	In-hospital Mortality	WBC, NC, LC, NLR	🟢⚪🟢🟢🟡🟢
Ge *et al.*[19]	2023	ATAAD	100	171	53	In-hospital Mortality	WBC, NC, LC, CRP	🟡⚪🟡🟢🟡🟢
Guo *et al.*[78]	2019	Mixed Cohort	39	109	52	In-hospital Mortality	CRP	🔴⚪🟢🟢🔴🟢
Guvenc and Engin[40]	2023	ATAAD	100	195	Survived=55, Deceased=59	In-hospital Mortality	WBC, NC, LC, NLR, CRP, NLPR	🟡⚪🟢🟢⚪🟡
Huang *et al.*[41]	2015	ATAAD	60.4	212	48.5	Mortality	WBC, CRP	🟡🟡🟡🟢🟡⚪
Huang *et al.*[20]	2023	ATAAD	100	247	Survived=61.39, Deceased=64.79	Mortality	WBC, CRP	🟡⚪🟢🟢⚪🟢
Kalkan *et al.*[4]	2017	ATAAD	100	184	53.1	In-hospital Mortality	NLR	🟡🟢🟢🟢⚪🟡
Karakoyun *et al.*[21]	2015	ATAAD	N/A	35	Survived=51.8, Deceased=67.8	In-hospital Mortality	WBC, NLR, CRP	🟡🟡🟢🟢⚪🟢

Ke *et al.*[22]	2021	ATAAD	100	118	50.11	In-hospital Mortality	WBC	🟡⚪🟡🟢⚪🟡
Keskin *et al.*[23]	2021	ATAAD	100	151	61	In-hospital Mortality	WBC, CRP	🟡⚪🟢🟢⚪🟢
Lafçi *et al.*[24]	2014	ATAAD	100	104	55.2	In-hospital Mortality	WBC, NC, LC, NLR	🟡⚪🟢🟢⚪🟡
Li *et al.*[81]	2015	ATAAD	35	106	48	Mortality	WBC, sTIPS	🔴⚪🟢🟡🔴🟢
Li *et al.*[89]	2016	ATAAD	67.9	103	55	In-hospital Mortality	CRP	🟡🟡🟢🟢⚪🟡
Li *et al.*[25]	2021	ATAAD	100	206	52	In-hospital Mortality	WBC, Nc, CRP	🟡⚪🟡🟢🟡🟡
Li *et al.*[82]	2022 (a)	ATAAD	99.80%	496	53.61	Mortality	WBC, NC, SII	🟢🟡🟢🟡⚪🟢
Li *et al.*[26]	2022 (b)	ATAAD	N/A	214	54.7	Mortality	WBC, NC, LC, CRP	🟢🟡🟡🔴⚪⚪
Lin *et al.*[42]	2019	ATAAD	81.9	536	Survived=52.94, Deceased=57.03	In-hospital Mortality	WBC, NC, LC, NLR, LMR, MC	🟢🟡🟢🟢🟡🟢
Liu *et al.*[27]	2017 (b)	ATAAD	100	251	49.02	30 Day Mortality	WBC, CRP	🟡⚪🟡🟡⚪🟡
Liu *et al.*[43]	2018	ATAAD	85.3	143	50	In-hospital Mortality	WBC, NC, LC, MC	🟡⚪🟡🟢🔴🟡
Liu *et al.*[77]	2020	ATAAD	100	55	N/A	Mortality	CRP	🔴⚪🟡🟡⚪🔴
Liu *et al.*[83]	2022 (a)	ATAAD	100	1967	54	90 Day Mortality	WBC, NC, NLR, PLR, CRP, Custom Index	🟢⚪🟡🟢⚪🟡
Liu *et al.*[84]	2022 (b)	ATAAD	100	2008	54	30 Day Mortality	WBC, NC, NLR, PLR, LMR, MC, Custom Index	🟢⚪🟡🟢⚪🟡
Liu *et al.*[85]	2022 (c)	ATAAD	100	Total=5014 (Training=3124, Internal Validation=571, External Validation=1319)	Training=50, Internal Validation= 49, External Validation=49	30 Day Mortality	WBC, NC, LC, NLR, PLR, Custom Index	🟢⚪🟢🟢⚪🟡
Liu *et al.* [86]	2022 (d)	ATAAD	100	2387	54	In-hospital Mortality	NC, PNR, Custom Index	🟡⚪🟡🟢⚪🟡

Ma *et al.*[48]	2020	ATAAD	100	331	48.3	In-hospital Mortality	WBC	
Ma *et al.*[44]	2023	ATAAD	100	879	Survived=54, Deceased=56	In-hospital Mortality	WBC, NC, LC, LMR, MC, EOS	
Oz *et al.*[28]	2017	ATAAD	100	57	Survived=53, Deceased=59	In-hospital Mortality	WBC, NC, LC, NLR	
Qin *et al.*[45]	2022	ATAAD	100	1180	53	In-hospital Mortality	WBC, NC, LC, CRP, MC, EOS	
Savas *et al.*[29]	2022	ATAAD	100	204	Survived=55.2, Deceased=60.6	In-hospital Mortality	WBC, NC, LC, CRP, MC	
Sbarouni *et al.*[75]	2015	ATAAD	100	120	63	In-hospital Mortality	NLR	
Shao *et al.*[46]	2022	ATAAD	56.3	183	Survived=49.53, Deceased=53.00	In-hospital Mortality	WBC, NC, LC, NLR, PLR, MC, EOS, LMR	
Song *et al.*[90]	2022	ATAAD	100	80	55.3	30 Day Mortality	CRP	
Suzuki *et al.*[49]	2020	ATAAD	100	466	normal WBCc=68.3, elevated WBCc=62.3	30 Day Mortality	WBC	
Vrsalovic *et al.*[30]	2015	Hypertensives with ATAAD	59	54	69	In-hospital Mortality	WBC, CRP	
Wen *et al.*[31]	2011	ATAAD	66.6	36	Survived=50.6, Deceased=67.4	In-hospital Mortality	WBC, CRP	
Xie *et al.*[80]	2021	ATAAD	100	270	PLR Tertile-1=50.93, Tertile-2=49.52, Tertile-3=49.52	In-hospital Mortality	LC, PLR	
Xu *et al.*[87]	2022	ATAAD	100	324	49.5	30 Day Mortality	Custom Index	
Xu *et al.*[32]	2023 (a)	ATAAD	N/A	320	51.8	In-hospital Mortality	WBC, NC, LC, NLR, PLR, Custom Index	

Xu *et al.*[73]	2023 (b)	ATAAD	100	410	49.1	In-hospital Mortality	NLR, Custom Index	amber, grey, green, green, grey, green
Yang *et al.*[33]	2020	ATAAD	N/A	520	50.1	In-hospital Mortality	WBC, NC, CRP	green, grey, green, green, grey, green
Yu *et al.*[72]	2023	Acute dissection, mixed	69.9	1903	53	In-hospital Mortality	LC	amber, amber, green, green, red, green
Zdravkovic *et al.*[34]	2020	ATAAD	100	116	60.8	In-hospital Mortality	WBC, NC, LC, NLR, CRP, EOS	red, grey, amber, green, grey, green
Zhang *et al.*[35]	2016	ATAAD	N/A	67	Survived=58.4, Deceased= 55.2	In-hospital Mortality	WBC, CRP	amber, grey, green, green, grey, amber
Zhang *et al.*[98]	2018	Chronic and Acute TAAD	81.78	999	49.8	In-hospital Mortality	WBC	amber, grey, amber, green, grey, amber
Zhang *et al.*[36]	2021 (a)	ATAAD	100	224	52.76	Mortality	WBC, NC, LC, NLR, CRP	amber, grey, green, green, amber, red
Zhang *et al.*[74]	2021 (b)	Acute dissection, mixed	44	179	55	In-hospital Mortality	NLR	amber, grey, green, green, grey, green
Zhao *et al.*[88]	2023	ATAAD	100	193	56	Mortality	Custom Index	amber, grey, amber, green, amber, amber
Zhou *et al.*[37]	2016	ATAAD	56	98	53.6	In-hospital Mortality	WBC, NLR, CRP	amber, grey, amber, green, grey, amber

ATAAD = acute type A aortic dissection, WBC = white blood cells, NC = neutrophil count or percentage, LC = lymphocyte count or percentage, MC = monocyte count, EOS = eosinophil count, NLR = neutrophil to lymphocyte ratio, PLR = platelet to lymphocyte ratio, LMR = lymphocyte to monocyte ratio, CRP = C reactive protein, sTIPS = simplified thrombo-inflammatory prognostic score.

QUIPS domains: 1 = study participation, 2 = study attrition, 3 = prognostic factor measurement, 4 = outcome measurement, 5 = study confounding, 6 = statistical analysis and reporting. QUIPS scoring: green = high, amber = moderate, red = low and grey = unknown or not applicable.

Thieme

Supplementary Table 4. Summary of study characteristics for studies using morbidity measures as a primary outcome measure.

Author	Year	Study Population	Operated (%)	Sample Size	Mean Age	Outcome of Interest	Extracted Biomarkers	QUIPS 1	2	3	4	5	6
Ge *et al.*[58]	2021	ATAAD - Debakey I	100	582	52.2	Prolonged mechanical ventilation	WBC, CRP	Green	Grey	Yellow	Yellow	Grey	Green
Guan *et al.*[59]	2023 (b)	ATAAD	100	77	48.2	Severe hypoxaemia	WBC, NC, CRP	Yellow	Grey	Yellow	Green	Grey	Green
Hua *et al.*[60]	2023	ATAAD	100	89	53.79	Post-Operative Pneumonia	WBC, NC	Yellow	Grey	Yellow	Green	Grey	Green
Jin *et al.*[61]	2017	ATAAD	100	121	46.6	Prolonged mechanical ventilation	WBC, CRP	Green	Green	Yellow	Yellow	Yellow	Green
Liu *et al.*[62]	2017 (a)	ATAAD	100	181	47.19	Hypoxemia	WBC	Yellow	Grey	Yellow	Green	Grey	Yellow
Sheng *et al.*[63]	2022	ATAAD	100	492	49.6	Severe Hypoxemia	WBC	Green	Grey	Yellow	Green	Yellow	Green
Wang *et al.*[71]	2022 (a)	ATAAD	100	479	51.96	Acute Lung Injury	NC	Yellow	Grey	Yellow	Yellow	Grey	Yellow
Wang *et al.*[64]	2022 (b)	ATAAD	100	492	49.6	Post-Op Tracheostomy	WBC	Yellow	Grey	Yellow	Green	Grey	Green
Wu *et al.*[65]	2020	ATAAD	100	79	51.8	Acute Lung Injury	WBC, CRP	Yellow	Grey	Green	Green	Grey	Yellow
Zhang *et al.*[66]	2023 (a)	Acute Aortic Syndrome	100	330	53.5	Oxygen Impairment	WBC, NC, LC, NLR, PLR, LMR, MC	Yellow	Grey	Yellow	Yellow	Grey	Green
Zhao *et al.*[67]	2021	ATAAD	100	64	No-ARDS=52.7, ARDS=50.6	Acute Respiratory Distress Syndrome	WBC	Red	Grey	Yellow	Green	Grey	Yellow
Zhou *et al.*[68]	2021	ATAAD	100	75	53.53	Hypoxemia	WBC	Yellow	Grey	Yellow	Yellow	Grey	Yellow
Chen *et al.*[70]	2022	ATAAD	100	159	52.9	Acute Kidney Injury	LMR	Green	Green	Green	Green	Grey	Green
Chen *et al.*[53]	2023(a)	ATAAD	100	255	No AKI=53.13, AKI=50.96	Acute Kidney Injury	WBC, NC, LC, MC, EOS	Green	Grey	Green	Green	Grey	Green
Dong *et al.*[50]	2020	ATAAD	100	326	53.4	Acute Kidney Injury	WBC	Green	Grey	Green	Green	Grey	Yellow

Guan *et al.*[51]	2023 (a)	ATAAD	100	106	No Stage III AKI=47.5, Stage III AKI=50.2	Stage III Acute Kidney Injury	WBC, NC	amber, grey, green, green, grey, green
Ko *et al.*[52]	2015	ATAAD	100	375	66.4	Acute Kidney Injury	WBC, CRP	green, amber, amber, green, grey, green
Ma *et al.*[79]	2021	ATAAD Debakey I	100	190	47	Acute Kidney Injury	LMR	amber, grey, amber, green, grey, amber
Zhang *et al.*[76]	2023 (b)	ATAAD	100	241	No AKI=47.53, AKI=51.06	Acute Kidney Injury	NLR, PLR	amber, grey, amber, amber, grey, amber
Guan *et al.*[54]	2018	ATAAD	100	126	49.1	Neurological complications	WBC	amber, grey, amber, amber, grey, green
Lin *et al.*[57]	2021	ATAAD	100	257	51.98	Delirium	WBC, NC, LC, MC	amber, grey, amber, amber, grey, amber
Lin *et al.*[55]	2023	ATAAD	100	146	53.69	Transient Neurological Deficit	WBC, CRP	amber, green, amber, amber, amber, amber
Tang *et al.*[69]	2022	ATAAD	100	199	Non-IAO=53.2, IAO=57.2	In-hospital adverse outcomes (IAO)	WBC, NLR, LMR, CRP	green, grey, amber, amber, amber, amber
Wang *et al.*[56]	2021	ATAAD	100	1476	50.83	Post-Operative headache	WBC	green, grey, amber, green, grey, green

ATAAD = acute type A aortic dissection, WBC = white blood cells, NC = neutrophil count or percentage, LC = lymphocyte count or percentage, MC = monocyte count, EOS = eosinophil count, NLR = neutrophil to lymphocyte ratio, PLR = platelet to lymphocyte ratio, LMR = lymphocyte to monocyte ratio, CRP = C reactive protein.

QUIPS domains: 1 = study participation, 2 = study attrition, 3 = prognostic factor measurement, 4 = outcome measurement, 5 = study confounding, 6 = statistical analysis and reporting. QUIPS scoring: green = high, amber = moderate, red = low and grey = unknown or not applicable.

Supplementary Table 5. Summary statistics from studies related to WBC counts in patients with postoperative morbidity.

	Author	Year	Without Outcome		With Outcome		p-value
			Number	WBC count	Number	WBC count	
**Kidney Injury**	Chen *et al.*[53]	2023(a)	131	11.01 ± 3.79	124	12.36 ± 4.47	0.009
	Dong *et al.*[50]	2020	104	9.6 (7.7-12.4)	222	11.9 (9.3-14.8)	<0.0001
	Guan *et al.*[51]	2023(a)	25	12.4 ± 3.7	81	11 ± 3.7	0.11
	Ko *et al.*[52]	2015	210	12.54 ± 4.69	165	12.94 ± 5.08	0.43
**Lung Injury**	Ge *et al.* [58]	2021	323	10.8 ± 3.8	259	11.6 ± 4.3	0.015
	Guan *et al.*[59]	2023(b)	49	10 ± 3.6	28	12.52 ± 3.4	0.005
	Hua *et al.*[60]	2023	61	11.44 ± 4.02	28	13.56 ± 4.72	0.032
	Jin *et al.*[61]	2017	86	9.15 ± 3.23	35	10.24 ± 3.88	0.115
	Lui *et al.*[62]	2017(a)	112	10.51 ± 2.72	48	13.82 ± 3.85	<0.0001
	Sheng *et al.*[63]	2022	373	9.5 (7.0-12.0)	119	12.0 (9.2-14.2)	<0.001
	Wang *et al.*[64]	2022(b)	437	9.8 (7.3-12.5)	55	12.1 (9.0-14.7)	0.001
	Wu *et al.*[65]	2020	41	11.01 ± 1.07	38	15.75 ± 3.86	<0.01
	Zhang *et al.*[66]	2023(a)	165	10.1 (7.2-13.05)	165	11.4 (8.7-14.28)	0.032
	Zhao *et al.*[67]	2021	28	9.84 ± 3.27	36	12.12 ± 3.43	<0.001
	Zhou *et al.*[68]	2021	39	10.5 ± 3.8	24	12.48 ± 3.97	0.014
**Neurological Injury**	Guan *et al.*[54]	2018	96	11.1 ± 3.2	30	13.5 ± 4.3	<0.01
	Lin *et al.*[57]	2021	154	11.8 ± 3.9	103	12.9 ± 4.1	0.033
	Lin *et al.*[55]	2023	88	12.1 ± 3.76	61	13.22 ± 3.49	0.069
	Wang *et al.*[56]	2021	756	9.97 (7.42-12.37)	277	10.45 (7.92-14.24)	0.002

Results are displayed as mean ± standard deviation or median and interquartile range.

Supplementary Table 6. Summary receiver operator characteristic analyses identified in the literature.

Author	Year	Outcome	Marker	AUC (CI)	Sensitivity	Specificity
Bedel *et al.*[5]	2019	In-hospital Mortality	PLR	0.75 (0.638, 0.882)	0.765	0.781
	2019	In-hospital Mortality	NLR	0.746 (0.623, 0.87)	0.706	0.768
Chen *et al.*[38]	2020	30-day Mortality	LNR	0.589 (0.543, 0.635)	0.69	0.45
	2020	30-day Mortality	LMR	0.575 (0.528, 0.621)	0.49	0.64
Chen *et al.*[70]	2022	AKI	LMR	0.719 (0.642, 0.787)	0.7021	0.6786
Dong *et al.*[50]	2020	AKI	WBC	0.642	0.387	0.837
Erdolu *et al.*[16]	2020	In-hospital Mortality	CRP	0.879 (0.81, 0.949)	0.75	0.58
	2020	In-hospital Mortality	NLR	0.835 (0.735, 0.934)	0.76	0.61
Feng *et al.*[17]	2017	Mortality	CRP	0.64 (0.574, 0.739)	0.7853	0.6823
Guo *et al.*[78]	2019	In-hospital Mortality	CRP	0.758 (0.667, 0.835)	0.9032	0.5513
Guvenc *et al.*[40]	2023	In-hospital Mortality	NLPR	0.739 (0.661, 0.817)	0.705	0.564
Huang *et al.*[20]	2023	Mortality	CRP	0.69 (0.62, 0.77)	0.5056	0.8671
Kalkan *et al.*[4]	2017	In-hospital Mortality	NLR	0.71 (0.631, 0.789)	0.71	0.63
Karakoyun *et al.*[21]	2015	In-hospital Mortality	NLR	0.829 (0.674, 0.984)	0.77	0.74
Lafci *et al.*[24]	2014	In-hospital Mortality	NLR	0.634 (0.516, 0.753)	0.70	0.53
Li *et al.*[81]	2015	Mortality	WBC	0.637	NA	NA
	2015	Mortality	sTIPS	0.743	NA	NA
Li *et al.*[89]	2016	In-hospital Mortality	CRP	0.70 (0.599, 0.789)	0.489	0.943
Li *et al.*[25]	2021	In-hospital Mortality	CRP	0.542 (0.468, 0.616)	0.704	0.411
	2021	In-hospital Mortality	WBC	0.641 (0.567, 0.71)	0.556	0.728
	2021	In-hospital Mortality	NC	0.653 (0.58, 0.721)	0.556	0.753
Li *et al.*[82]	2022	In-hospital Mortality	SII	0.61 (0.53, 0.7)	NA	NA
Lin *et al.*[42]	2019	In-hospital Mortality	LMR	0.849 (0.81, 0.888)	79.1	80.5
Liu *et al.*[83]	2022a	90-day Mortality	SCI	0.656 (0.615, 0.697)	NA	NA
	2022a	90-day Mortality	CRP	0.583 (0.542, 0.623)	NA	NA
	2022a	90-day Mortality	WBC	0.578 (0.537, 0.620)	NA	NA
	2022a	90-day Mortality	PLR	0.569 (0.527, 0.610)	NA	NA
	2022a	90-day Mortality	NLR	0.476 (0.439, 0.513)	NA	NA
	2022a	90-day Mortality	SII	0.522 (0.482, 0.562)	NA	NA
Liu *et al.*[85]	2022c	30-day Mortality	WBC	0.628 (0.584, 0.673)	NA	NA
	2022c	30-day Mortality	NC	0.568 (0.521, 0.614)	NA	NA
	2022c	30-day Mortality	LC	0.511 (0.468, 0.554)	NA	NA
	2022c	30-day Mortality	PLR	0.558 (0.512, 0.604)	NA	NA

	2022c	30-day Mortality	NLR	0.503 (0.46, 0.546)	NA	NA
	2022c	30-day Mortality	STI	0.664 (0.62, 0.708)	NA	NA
Liu *et al.*[86]	2022d	In-hospital Mortality	PNR	0.611 (0.577, 0.644)	NA	NA
	2022d	In-hospital Mortality	NC	0.598 (0.563, 0.634)	NA	NA
	2022d	In-hospital Mortality	5A	0.736 (0.7, 0.771)	NA	NA
Ma *et al.*[48]	2020	In-hospital Mortality	WBC	0.64 (0.6, 0.68)	0.736	0.55
Oz *et al.*[28]	2021	In-hospital Mortality	NLR	0.919 (0.832, 1)	0.86	0.91
Song *et al.*[90]	2022	30-day Mortality	CRP	0.771 (0.66, 0.882)	NA	NA
Tang *et al.*[69]	2022	In-hospital adverse events	CRP	0.6	0.302	0.904
Vrsalovic *et al.*[30]	2015	In-hospital Mortality	CRP	0.79	0.83	0.8
Xu *et al.*[87]	2022	30-day Mortality	SII	0.712 (0.638, 0.787)	NA	NA
Xu *et al.*[76]	2023b	In-hospital Mortality	SIRI	0.718	NA	NA
	2023b	In-hospital Mortality	NLR	0.692	NA	NA
Zdravkovic *et al.*[34]	2020	In-hospital Mortality	CRP	0.702	0.714	0.75
Zhang *et al.*[74]	2021b	In-hospital Mortality	NLR	0.695 (0.619, 0.795)	0.905	0.5
Zhou *et al.*[37]	2016	In-hospital Mortality	CRP	0.69 (0.51, 0.88)	0.8	0.69

AKI = acute kidney injury, PLR = platelet to lymphocyte ratio, NLR = neutrophil to lymphocyte ratio, LNR = lymphocyte to neutrophil ratio, LMR = lymphocyte to monocyte ratio, WBC = white blood cells, CRP = C reactive protein, NLPR = neutrophil/lymphocyte/platelet ratio, sTIPS = simplified thrombo-inflammatory score, NC = neutrophil count or percentage, SII = systemic immune-inflammation index, SCI = systemic coagulation-inflammation index, LC = lymphocyte count or percentage, STI = systemic thromboinflammatory index, PNR = platelet to neutrophil ratio, 5A = 5A custom model, SIRI = systemic inflammatory response index.
